# Inclusion of Oat and Yeast Culture in Sow Gestational and Lactational Diets Alters Immune and Antimicrobial Associated Proteins in Milk

**DOI:** 10.3390/ani11020497

**Published:** 2021-02-14

**Authors:** Barry Donovan, Aridany Suarez-Trujillo, Theresa Casey, Uma K. Aryal, Dawn Conklin, Leonard L. Williams, Radiah C. Minor

**Affiliations:** 1 Department of Animal Sciences, North Carolina A&T State University, Greensboro, NC 27411, USA; bcdonova@gmail.com (B.D.); drconkli@ncat.edu (D.C.); 2 Department of Animal Sciences, Purdue University, West Lafayette, IN 47907, USA; asuarezt@purdue.edu (A.S.-T.); theresa-casey@purdue.edu (T.C.); 3 Department of Comparative Pathobiology and Purdue Proteomics Facility, Purdue University, West Lafayette, IN 47906, USA; uaryal@purdue.edu; 4 Center for Excellence in Post-Harvest Technologies, North Carolina A&T State University, Kannapolis, NC 28081, USA; llw@ncat.edu

**Keywords:** immunoglobulin, milk, proteomics, post-weaning, lactation, piglets, sows, supplementation, oat, yeast culture

## Abstract

**Simple Summary:**

This study investigated the impact that supplementing sow’s gestation and lactation feed with oat alone or together with brewer’s yeast has on milk proteins and piglet growth and health. Oat and yeast supplements increased abundance of several milk proteins involved in immune protection. Piglets born from either the oat- or yeast-supplemented sows had decreased incidence of diarrhea after weaning. The average birth weights for piglets born of dams that consumed Oat were significantly greater than those that did not. However, piglets born to sows that consumed yeast in combination with oat weighed less at weaning and gained the least amount of weight post-weaning. These data suggest that oat, and to a lesser extent, yeast, added to maternal diets during gestation and lactation can positively impact milk, growth, and health of offspring but given in combination can potentially negatively affect piglet weight gain.

**Abstract:**

Maternal diet supplementation with pro- and prebiotics is associated with decreased incidence of diarrhea and greater piglet performance. This study investigated the impact adding whole ground oat as a prebiotic, alone or in combination with a probiotic, yeast culture (YC) (*Saccharomyces cerevisiae*), to sow gestation and lactation rations had on milk protein composition, piglet growth, and incidence of post-weaning diarrhea (PWD). Diets: control (CON), CON + yeast culture (YC) [5 g/kg], CON + oat (15% inclusion rate) (Oat) or CON+ YC [5 g/kg] + Oat (15%) were fed the last 30 days of gestation and throughout lactation (18–21 days). Shotgun proteome analysis of day 4 and 7 postpartum milk found 36 differentially abundant proteins (P-adj < 0.1) in both Oat and YC supplemented sows relative to CON. Notable was the increased expression of antimicrobial proteins, lactoferrin and chitinase in milk of Oat and YC sows compared to CON. The levels of IgA, IgM (within colostrum and milk) and IgG (within milk) were similar across treatments. However, colostral IgG levels in Oat-supplemented sows were significantly lower (*p* < 0.05) than that of the control sows, IgG from Oat-supplemented sows displayed greater reactivity to *E. coli*-antigens compared with CON and YC. Piglets from sows that consumed Oat alone or in combination weighed significantly more (*p* < 0.05) at birth compared to CON and YC. However, piglets in the Oat + YC group weighed less at weaning and had the lowest weight gain (*p* < 0.05) postweaning, compared with CON. Taken together with the observation that piglets of either YC- or Oat-fed sows had less PWD compared to CON and YC+ Oat suggests that Oat or YC supplementation positively impacts piglets through expression of certain milk-associated immune and antimicrobial proteins.

## 1. Introduction

Pork is the most consumed animal globally. In 2018, pork accounted for over 40% of the world meat/poultry consumption [1] and despite the recent losses stemming from the novel coronavirus pandemic, the industry remains critical to global meat production and economy [2]. To keep pace with the demand for pork, producers often wean piglets between one to three weeks of age [3]. This practice is often associated with anorexia, growth retardation, morbidity, and mortality. While post-weaning mortality can be attributed to a variety of reasons, the number one disease-related cause of death is post-weaning diarrhea (PWD) [4,5,6].

Piglets weaned before three weeks of age are particularly susceptible to PWD because they lack a fully matured immune system [7]. Moreover, the stressors introduced by the weaning process, such as separation from their dams and littermates, and changes in environment and nutrition [8,9,10], further weaken an immature immune system [5,11,12,13,14]. Immuno-incompetence of the piglet is further exacerbated because passive immunity provided by milk is halted upon weaning [15]. These conditions coupled with an immature intestinal tract, lead to microbial dysbiosis within the gut of the young piglet and increase susceptibility to colonization by enteric pathogens [4,10,16,17].

In addition to providing nutrients to support the growth of neonates, colostrum, and milk [18,19] are also a source of cellular and non-cellular immune components, antibacterial factors [20,21,22,23], and other bioactive compounds [24,25,26]. These milk components function to promote intestinal health, protect the neonate against infection, modulate immune responses, drive the development of the enteric nervous system, and stimulate the establishment of the gut microbiome [18,27,28,29,30,31]. Milk composition can be altered by maternal (gestational and lactational) diets. The changes in milk composition can ultimately impact growth, immunity, and gut health of offspring [32,33,34,35,36,37,38], and so investigations of how supplementation of sow’s diets during gestation and lactation impact piglet performance are receiving considerable attention [39,40,41,42,43].

Sow diets supplemented with probiotics or prebiotics were shown to decrease piglet diarrhea and mortality rates, and lead to greater weaning weights [44,45]. The positive impact may be partially attributed to changes in milk components. For example, milk of sows fed a live yeast (*Saccharomyces cerevisiae*) supplement during lactation had elevated IgG levels in colostrum, which resulted in higher levels of plasma IgG in their progeny at 24 h postnatal [46,47]. Supplementation with dry yeast, yeast cell wall or purified (1,3)-(1,6)-β-D-glucan from yeast has been shown to impact milk and piglet performance [48,49,50]. Furthermore, incorporation of oat hulls as a prebiotic source into sow gestation feed increased total litter weights [51,52] and 15% oat supplementation into sow gestational and lactational feed increased levels of bifidobacteria in milk [53]. In addition, both *Saccharomyces cerevisiae* and oat have been shown to impact immune responses and associated components [54,55]. Thus, we were interested in comparing the impact of these supplements on immune associated components in the milk when given separately as well as in combination. The hypothesis driving this work is that inclusion of oat or yeast culture (*Saccharomyces cerevisiae*) separately or in combination as additives in sow’s gestation and lactation ration will lead to changes in milk protein composition and influence piglet weight and incidence of PWD.

## 2. Materials and Methods

### 2.1. Animals, Housing and Care

This study was conducted after approval by the Institutional Animal Care and Use Committee of North Carolina A&T State University protocol number 10.003.0. All animals in this study were reared, housed, and maintained at the North Carolina A&T State University Swine Research Indoor Unit and subject to routine-health farm management protocols, which included vaccinating and administration of antibiotics following veterinary directives, if required. A total of sixteen sows of mixed lineage (either Landrace x Yorkshire or Berkshire) of second—forth parity and an average weight of 189 kg ± 39.2 (mean ± SD) were artificially inseminated using semen from the same sire (Choice Genetics, LLC. NC Branch). For the study, sows were randomly divided into four experimental groups (*n* = 4 in each group) and housed in individual gestation crates; after farrowing, sows remained in gestation crates with nursing piglets. The average birth litter size was 10.6 ± 2.96 (mean ± SD), with 8.63 ± 2.45 (mean ± SD) born alive. At 18–21 days of age, a total of 120 piglets were weighed and weaned. A subset of weanlings, 80 piglets (*n* = 20 per maternal diet, including female and castrated males) with weights closest to the average weaning weight 6.42 kg ± 1.69 (mean ± SD), were selected and assigned to a nursery pen (*n* = 5 per pen) based on the feed their dams received. There were four pens per diet group, divided between two nursery rooms to ensure that each diet group was represented twice in each room.

### 2.2. Feed Administration

All feed was milled at the North Carolina State University Feed Mill and was formulated to meet or exceed the nutritional needs of the sows according to the National Research Council [56]. Four dietary treatments were used during both the gestation and lactation periods and were as follows: (1) corn- and soy-based control diet [without antibiotic growth promoters (CON)], (2) control + live yeast culture (YC) (*S. cerevisiae*) 5 g/kg of ration (Diamond V Mills, Inc., Cedar Rapids, IA, USA), (3) control + ground whole oat (15% inclusion into ration), and (4) control + live YC 5 g/kg of ration + ground whole oat 15%. The gestation feed (Table 1) was administered beginning on the 85th day of gestation and ending on day of farrowing. The lactation feed (Table 2) was administered beginning on day of farrowing and ending on the day piglets were weaned (18–21 days). Prior to the administration of the experimental diets, all sows were fed the CON gestation diet and sows received the same feed supplementation during gestation and lactation. Upon weaning, all piglets were fed the same nursery ration (Table 3), formulated to meet all nutritional requirements [56]. All animals had ad libitum access to feed and water. Nutrient composition of diets was not determined.

### 2.3. Piglet Feed Intake and Weight Measurements

Piglets were fed daily and had ad libitum access to ration (Table 3) and water. Nutrient composition of piglet ration was not determined. The average feed intake for the two-week post-weaning period was calculated for each of the 16 pens by subtracting the amount of feed left from the total amount of feed offered. Piglet weights were recorded at birth, day of weaning, and days 7, 14, 21 and 28 post-weaning.

### 2.4. Fecal Scoring

Observations of fecal consistency were made and recorded on the day of weaning (day 0), and days 7 and 14 post-weaning. Seven independent reviewers that were blinded to treatment, visually evaluated and scored the consistency of fecal matter from each of the 5 piglets in each pen using a scoring rubric where (1) firm pellets, (2) normal pellets, (3) soft pellets, (4) soft without pellets but not runny, (5) runny.

Average fecal scores (*n* = 7) for each of the four diets were calculated. To generate the average diet score, the fecal scores score of each piglet (*n* = 5) in the pen were used to generate an average pen score for each diet (*n* = 4 pens/diet), the pen scores were then used to generate an average for the dietary treatment.

### 2.5. Milk Collection

On day of farrowing (day 0), and days 1, 4, 7, and 14 of lactation, oxytocin (10 IU in 1.0 mL) was injected intramuscularly, 10 min later, colostrum and milk were manually expressed from all functional glands into sterile 50 mL conical tubes. Samples were stored at −80 °C until analyzed.

### 2.6. Milk Immunoglobulin and Proteome Analysis

Total porcine immunoglobulin (IgG, IgM, and IgA) concentrations were determined by ELISA (Bethyl Labs, Montgomery, TX, USA) following the manufacturer’s protocol. To compare *E. coli*-specific IgG in colostrum, extracts from *E. coli* (cultured from sow feces) were prepared using B-PER Bacterial Protein Extraction Reagent (Thermo Fisher Scientific, Waltham, MA, USA). Extracted bacterial proteins were separated by SDS-PAGE with a 10% 2- well IPG/prep gel (cat. Number 4561031, Bio-Rad, Hercules, CA, USA) and transferred onto PDVF membrane, blocked (5% low fat cow milk), and probed with 100 μL of sow colostrum (day 0) diluted 1:2 in PBS in a surf-blot system. The blot was probed with anti- IgG-HRP [1:1000] (Bethyl Labs, Montgomery, TX, USA) for 1h at room temperature. The image was generated with ChemiDoc™ MP Imaging System (Bio-Rad, Hercules, CA, USA).

Gel-free and label-free quantitative proteomic analysis of homogenized porcine milk samples for CON, YC and Oat (because they were associated with positive impacts to piglets) were done using shot-gun liquid chromatography tandem mass spectrometry (LC–MS/MS) at the Purdue Proteomics Facility, Bindley Bioscience Center, Purdue University. Equal volumes of milk (0.5 mL each) collected on day 7 and 14 of lactation from sows fed control (*n* = 2), oat diet (*n* = 3) and yeast diet (*n* = 3) were homogenized at 35,000 psi using a Barocycler (4 °C, 60 cycles: 50 s at 35,000 psi and 10 s at ATM) to release proteins and other molecules from extracellular vesicles including exosomes. Then, proteins were extracted by the addition of a 3-fold volume of 8 M urea to the sample as a denaturing agent. This method separates fat from fat-soluble casein proteins in addition to whey proteins. Following extraction, proteins were precipitated using 4-fold volume of cold (−20 °C) acetone overnight. Precipitated proteins were pelleted by centrifugation at 13,500 rpm for 15 min at 4 °C, and dissolved in 8M urea containing 10 mM dithiothreitol and incubated at 37 °C for 1 h for reduction followed by alkylation using alkylating reagent (195 µL acetonitrile, 1 µL triethylphosphine and 4 µL of iodoethanol) and incubated for 1 h at 37 °C. The trypsin/LysC mix (20 µg) was dissolved in 400 µL of 25 mM ammonium bicarbonate, and 80 µL was added to each sample for digestion. Digestion was performed at high pressure using a Barocycler (50 °C; 60 cycles: 50 s at 20k PSI and 10 s at 1 ATM). Digested peptides were desalted using MicroSpin columns (C18 silica; The Nest Group), dried 5-fold volume of cold (−20 °C) acetone, trypsinized and analyzed in the Q Exactive Orbitrap HF mass spectrometer coupled with the Dionex UltiMate 3000 RSLC nano System as described previously [57,58,59]. Briefly, reverse-phase peptide separation was accomplished using a trap column (300 μm ID × 5 mm) packed with 5 μm 100 Å PepMap C18 medium coupled to a 50 cm long × 75 µm inner diameter analytical column packed with 2 µm 100 Å PepMap C18 silica (Thermo Fisher Scientific). Peptides were separated in the analytical column at a flow rate of 300 nL/min using a 120-min LC gradient. LC–MS data were collected using an HCD fragmentation scheme at a resolution of 120,000 at 200 *m*/*z* precursor ions, and at a resolution of 15,000 at 200 *m*/*z* for MS/MS ions. MS data were acquired in Data Dependent Acquisition mode. Reverse-phase LC–ESI–MS/MS was done as previously described [57]. Analysis of raw LCMS/MS was done with MaxQuant software (v. 1.6.0.16) [60].by searching MS/MS spectra against the Uniprot *Sus Scrofa* protein database using trypsin and LysC enzyme specificity and limited to ≤2 missed cleavages. Variable and fixed modifications were defined as oxidation of methionine and N-terminal acetylation, and iodoethanol of cysteine, respectively. The minimum amino acid length was set at 7, with a 10 ppm precursor mass tolerance, and 20 ppm MS/MS fragment ion tolerance. Peptide quantitation was performed using ‘unique plus razor peptides’, and the false discovery rate (FDR) was set at 0.01 for both peptide and protein identification. Label-free quantification (LFQ) intensities were used to calculate relative protein abundance, and data searched with “match between runs” option. Proteins matching to the reverse database were removed. Proteomic data were made publicly available by depositing in MassIVE and can be accessed with the following reference ID No. MSV000086562.

### 2.7. Statistical Analysis

GraphPad Prism v9.0 was used to create and perform statistical analysis for all graphs. For all graphs, a one-way analysis of variance (ANOVA) or a 2-way repeated measures analysis of variance (ANOVA) in case of post-weaning weights were used. The statistical analysis of LC–MS/MS search results was conducted using the InfernoRDN (Supplemental Table S1 and Supplemental Figure S1) software that uses R script for the statistical tests. Differentially abundant proteins were identified by two-way analysis of variance (ANOVA), and Tukey adjustment using protein LFQ values. Proteins were considered differentially abundant between control and treatment at P-adj < 0.1. Functional annotation analysis of proteins was done using Database for Annotation, Visualization and Integrated Discovery (DAVID) v6.8 [61,62], GeneCards [63] and Uniprot [64] databases were used to define protein function. Proteins were considered differentially abundant at P-adj < 0.1.

## 3. Results

### 3.1. Piglet Weights

**Figure 1:**
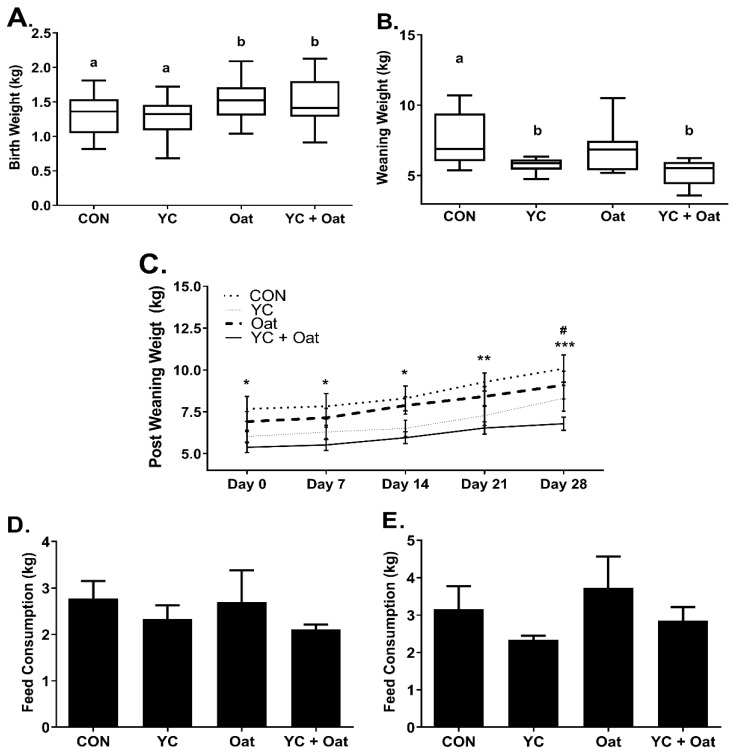
Piglet Weights and Post-Weaning Feed intake. Piglet weights (*n* = 20) were taken on (**A**) day of birth and (**B**) day of weaning. a,b Different letters indicate a significant difference at *p* < 0.05. (**C**) Weights starting on day of weaning d0 and including days, 1, 7, 21, and 28 post-weaning. Data are an average of *n* = 10. Statistical difference between the CON and YC + Oat groups * *p* < 0.05, ** *p* < 0.01 *** *p* < 0.001. (**#**) Statistical difference between Oat and YC + Oat *p* < 0.05. Feed intake (**D**) for first week and (**E**) second week post-weaning. Data are an average of (*n* = 4).

Piglets born to dams in the Oat and the Oat + YC group had an average birth weight of 1.54 ± 0.3 kg (mean ± SD) and 1.51 ± 0.3 kg (mean ± SD), respectively, which was significantly (*p* < 0.05) heavier than piglets born to the CON 1.27 ± 0.27 kg (mean ± SD) or YC 1.26 ± 0.26 kg (mean ± SD) supplemented sows (Figure 1A). Whereas on day of weaning, there were no weight differences between Oat 6.86 ± 1.8 kg (mean ± SD) and CON 7.66 ± 1.9 kg (mean ± SD) (Figure 1B) piglets. At weaning, piglets in the YC 5.77 ± 0.5 kg (mean ± SD) and the YC+ Oat 5.25 ± 0.9 kg (mean ± SD) groups weighed significantly (*p* < 0.05) less than CON. Piglets in the YC+ Oat group continued to weigh significantly less than piglets in the CON group throughout the 4-week post-weaning period (Figure 1C) despite no difference in post-weaning feed intake (Figure 1D,E).

### 3.2. Post-Weaning Diarrhea Scores

**Figure 2:**
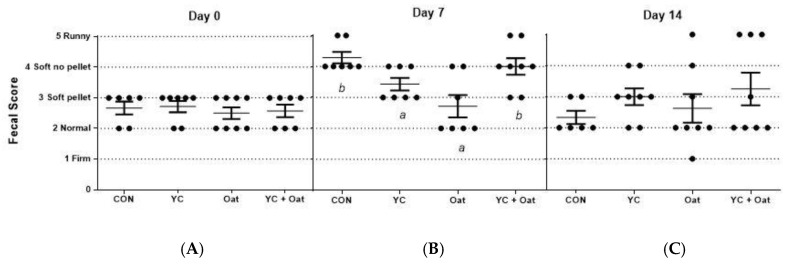
Fecal Scoring. Fecal samples were scored for diarrhea by seven participants blinded to treatments. Each visually scored the fecal matter using the following scoring criteria; (1) firm fecal pellets (2) normal pellets (3) soft pellets (4) soft no pellets but not runny (5) runny. The average scores (**A**) on day of weaning (day 0), (**B**), day 7 post-weaning, and (**C**) day 14 post-weaning are graphed. Data are an average of *n* = 7. Differences between letters *a* and *b* = *p* < 0.05.

Incidence of diarrhea was monitored for the first fourteen days of the post-weaning period. On day seven post-weaning the incidence of PWD was lower in the piglets born to sows given either the.15% oat or the YC supplemented feed as compared to those born to sows given either the control or the yeast culture + oat supplemented diets (Figure 2).

### 3.3. Milk Immunoglobulins

**Figure 3:**
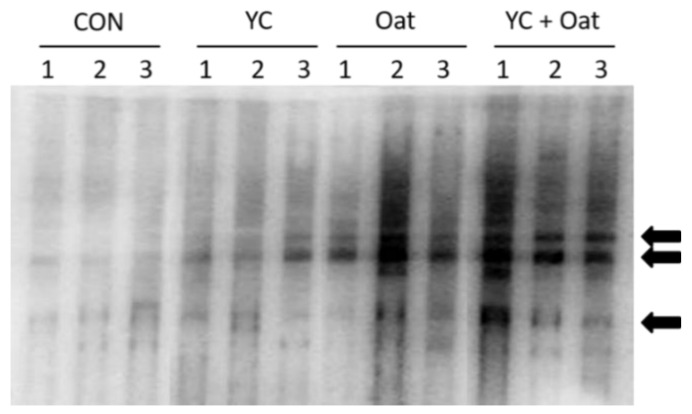
Milk IgG Reactivity. The *E. coli* specific reactivity of milk IgG was determined after probing resolved and immobilized *E. coli* extracts with milk collected from sows (*n* = 3) in each diet group: control (CON), yeast culture (YC), and Oat. Arrows indicate major reactivity with *E. coli* antigens.

IgA and IgM content of colostrum samples was similar across all diet groups on all days sampled. In colostrum samples, IgG levels were significantly (*p* < 0.05) lower in the 15% Oat fed group 6.0 ± 3.1 (mean ± SD) compared to the YC group 14.7 ± 2.1 (mean ± SD) (Figure 3). Milk IgA, IgM, and IgG content on days 4, 7, and 14 of lactation were similar (Table 4).

### 3.4. Immunoglobulin Reactivity

IgG within the milk of the Oat supplemented sows and to a lesser extent YC supplemented sows had increased reactivity to *E. coli* antigens (Figure 3).

### 3.5. Milk Proteome Analysis

There were 332 proteins detected in at least one of the treatment groups, of which 255 proteins were commonly expressed across all groups (Table S1). The 255 commonly expressed proteins were submitted to DAVID for functional annotation analysis and 214 were mapped to known DAVID IDs. The two most highly enriched categories within the Gene Ontology (GO) Biological Process were platelet degranulation and negative regulation of endopeptidase activity (Table 5).

Proteins within platelet aggregation included complement cascade proteins (C3, CFI and C4), five serpin proteins (SERPIN A, C, D, F and G), three fibrinogens (FGA, FGB and FGG) and plasminogen. Proteins categorized as negative regulation of endopeptidase activity included seven serpins (SERPIN A1, A2, A3, B, C, D, F1, F2 and G), three inter-alpha-trypsin inhibitors (ITIH1, 2 and 4), A2M, AMBP and WAP. Categories enriched within GO Cellular Component included extracellular exosome, extracellular space, and blood microparticle. The two first terms were enriched with proteins traditionally found outside the cell or as a product of cellular secretion including multiple milk proteins (CSN1S1, CSN1S2, CSN2, CSN3, LALBA, LTF, MFGE8, LPO and ALB). The blood microparticle term was enriched by multiple proteins present in the blood, such as hemoglobin (HBA1 and HBB), fibrinogen (FGA, FGB and FGG), FN1, PLG, TF, complement proteins (C3 and C4A), GC and several apolipoproteins (APOA1, A4 and E). The KEGG pathway highly enriched with commonly expressed proteins was complement and coagulation cascades (Table 5).

There were 29 high-confidence proteins (expressed in at least 2 samples/group) differentially abundant in milk samples between treatments (P-adj < 0.1; Table 6). Three proteins were more than twice the abundance of CON in milk of YC and Oat supplemented sows. Those proteins were cholinesterase (BChE), serpin family D member 1 (SERPIND1) and chitinase domain-containing protein 1 precursor (CHID1). CES1, LTF, AHSG, RAB7A and PPIC were also more abundant in milk of sows in the YC and Oat groups. Proteins less abundant in milk of YC and Oat sows compared to CON were related to protein synthesis (RPLP0, RPS7, RPSA, RPLP2, HSP90B1, HSP90AA1 and PDIA3), translation (EIF5A, EEF2 and RNASE4), cytoskeleton (TUBA1A and ACTN4) and energy production (VCP, MDH2, CKB and ATP5B).

## 4. Discussion

Inclusion of prebiotics, probiotics alone or in combination (synbiotics), in swine maternal diets has been shown to help limit the occurrence of gastrointestinal diseases and disorders in neonates [44,65]. The goal of this study was to investigate how inclusion of whole-ground oats alone or in combination with yeast culture (*Saccharomyces cerevisiae*) into sow gestation and lactation diets impacted piglet growth and incidence of post-weaning diarrhea. The central findings are that supplementation with Oat led to increased birth weights, maintenance of weight post-weaning, and significantly lower rates of post-weaning diarrhea compared to CON and YC alone. Furthermore, the combination of YC and Oat appeared to have a negative effect on growth. Analysis of milk suggests that the factors contributing to better growth and lower incidence of PWD in piglets born of the Oat supplemented sows may have been influenced by alternations in expression of immune associated and antibacterial proteins.

Oats are a good source of fiber. Inclusion of fiber in gestation and lactation diets has varying effects on offspring [66]. Supplementation of sows’ gestation diet with oat (41.5%) and oat hulls (51.2%) was reported to result in lower total litter birth weights and reduced growth rates between 3 days and 8 weeks of age [52]. In contrast, others found piglets born to sows that consumed oat hulls in gestation feed had increased total litter birth weight compared to control; and that litter weight gain during the 3-week pre-weaning period trended to be higher than control group [51]. Here, we report that inclusion of Oat lead to increased piglet birth weight and maintained postnatal growth compared with CON and YC.

The findings reported here that YC supplementation of sows does not result in increased birth weights is consistent with the literature [48,67,68]. Whereas some report, as we do here, no significant changes in weaning weights [50,68], others report that piglets born to dams given a YC supplement, compared to control, weighed more [45,67] or less at weaning [48]. Yeast culture supplementation has been reported to lead to increased piglet weight gain during the post-weaning period [48,50,68], which contrasts with our findings. The variations between our observations and those reported by others may be influenced by differences in experimental design; including the types of yeast formulation used, the inclusion method and rate, the initiation and length of supplementation and whether the piglets consumed the supplements as a creep feed prior to weaning. Post-weaning, all piglets were provided the same amount and formulation of feed and no significant differences in feed intake was observed and thus was not a major contributor to the variations in growth observed in this study.

Piglets that acquire post-weaning diarrhea have decreased growth rates. Here, we report differences in incidences of PWD that align with the post-weaning growth rates. For example, piglets in Oat group had lowest incidence of PWD and the least amount of weight loss post-weaning compared to all other groups. Interestingly, when Oat and YC were combined the positive effect was lost. At weaning, piglets born to the Oat + YC group weighed significantly less than CON and gained the least amount of weight during the 4-week post-weaning period. The mechanism for this is unknown, but milk compositional changes are a possible explanation. Both oat and yeast supplementation during gestation and lactation are associated with increased milk production and/or changes in protein and fat content of milk of dairy cattle [69,70,71]. In addition, YC and Oat supplementation of sows may influence the types of probiotic bacteria and associated metabolites expressed within milk, which, ultimately, could have antagonistic effects on the piglet gut [72]. While some report that supplementation with YC does not result in changes in milk composition [46,73], others demonstrate significant increases in total solids and crude protein or fat within sow milk [48]. Thus, differences in milk quantity and/or quality could be attributed to the effects observed in this study. Comparisons of milk quantity and quality were not investigated.

Both yeast and oat contain beta-glucan. Beta-glucan extracted from the cell wall of yeast, *Saccharomyces cerevisiae* ((1,3)-(1,6)-β-D-glucan) is a biological response modifier that modulate immune responses and growth of pigs [54,74,75]. Oats are also a rich source of β-D-glucan but with different linkages. A study by Estrada et al., reported that oat beta glucan restored immune responses (lymphocyte proliferation and IgG production) in animals treated with dexamethasone in comparison to healthy non-dexamethasone-treated control [55]. Moreover, piglets are born deprived of antibodies or agammaglobulinemic and thus depend on passive immunity from immunoglobulins within colostrum and milk to provide protection from viral and bacterial infections during the preweaning period [21,22,23]. This prompted us to investigate the impact that YC and Oat make on certain the levels of immunoglobulins within the milk. The data reported here show that 15% oat supplementation had no impact on the expression levels of IgM or IgA in colostrum or milk, but compared to the YC supplemented group, there was significantly less colostrum IgG. While some report that the IgG concentration within colostrum is higher in yeast supplemented sows compared to control [46,47,48]; others find that sows fed brewer’s yeast hydrolysate or YC as a supplement during gestation have no changes in IgG, IgM, or IgA in colostrum or milk [45,73]. Once again, the differences in these data could be attributed to the type of yeast culture product, the length and rate of inclusion, and the level of β-D-glucans (which was not determined). Furthermore, vaccinations or pathogenic exposures that may have occurred during the time of the studies could contribute to changes in the expression of antibodies by the sows and is an aspect that has not been addressed in these studies.

Whereas immunoglobulin A (IgA) is a key protective mediator of mucosal surfaces and essential to mitigating development of PWD [76,77], a role for IgG in the intestine has been described. IgG in milk functions to protect neonate against systemic and enteric pathogen exposures [21,77] and in humans, maternal IgG passed via milk to sucking infants provides protection from pathogens [78]. We report that although low, the IgG within the colostrum of the Oat (and to a lesser extent the YC) supplemented sows had increased reactivity to *E. coli* antigens as compared to control. Consistent with this finding, Werner et al. reported greater concentrations of IgG with specificity for *E. coli* LPS within the blood of piglets born to sows that consumed Jerusalem artichoke as a fiber source [79]. With these data taken together, we hypothesize that the increase in *E. coli*-specific IgG detected in colostrum of sows that consumed oat in this study may be contributing to the immune protection that led to the decreased incidence of PWD and maintenance of post-weaning weight.

Proteomic analysis of the milk was done to further investigate the impact that inclusion of YC and Oat singularly during gestation and lactation had on protein expression in milk. We report that milk collected from the sows showed commonly expressed proteins like those reported from other analyses of pig milk [80]. Functional annotation analysis of commonly expressed proteins found protein-enriched terms related with the secretion of milk components, but also with components in the blood (coagulation and complement cascade proteins). Although only five proteins were clustered in the GO Biological Process Lactation (CSN2, CSN3, RPLP0, SERPINC1 and XDH), further exploration of proteins found multiple milk proteins (CSN1S1, CSN1S2, LGB, LTF, LPO, ALB, MFGE8, FABP3, IGHG and WAP).

Proteins differentially abundant in milk of YC and Oat sows versus milk from CON animals were similar. Proteins less abundant in milk of YC and Oat supplemented sows were related to regulation of protein synthesis. Proteins more abundant in milk of oat and yeast culture supplemented sows may have provided some of the growth advantage evident in suckling neonates, to include, SERPIND1, which is a protease inhibitor. Milk is enriched with proteases and proteases aid with both, neonate’s digestion, and the release of bioactive peptides. Protease inhibitors such as SERPIND1 help to balance the activity of proteases and prevent premature and controlled degradation of milk proteins [81]. Furthermore, in the milk of supplemented sows butyrylcholinesterase (BChE) was more abundant. BChE functions to hydrolyze choline-based esters and is important in the detoxification of multiple neurotoxic molecules and was reported by others to be ten times higher in pig milk than in serum [82].

Lactoferrin (LTF), a normal component of milk and colostrum, which hyrdrolyzes RNA molecules, was also more abundant in milk of both yeast and oat supplemented sows. LTF functions to facilitate binding and transporting iron ions and has antibacterial, antiviral, and antiparasitic properties [83,84]. Enteral supplementation of preterm human infants with LTF was shown to reduce the effects of enteritis in neonates [85]. Thus, the improvements in piglet stool scores of supplemented groups observed in this study may be due to the higher content of LTF in milk. Alpha-2-HS-glycoprotein (AHSG) is present in the milk fat globule membrane [86] that promotes endocytosis and functions in calcium and lipid transport [87,88]. The chitinase enzyme (CHID1) was also more abundant in milk of both yeast and oat supplemented sows. As chitinase breaks down chitin, a protein found in the exoskeleton of insects, fungi, yeast, and algae [89], the increase of CHID1 could help piglets fight against the colonization of the intestines with pathogenic fungi or yeast. These data demonstrate that there are differentially expressed proteins associated with digestion and protection from pathogens that may explain some of the better performance of offspring born to dams in Oat and YC supplemented groups.

There are several limitations to this study. To begin, the level of sow feed intake and weight, or body condition in relation to Oat and YC supplements were not investigated. Additionally, the litter sizes produced by our research sows, while being within the expected range of smaller US operations [90], had fewer piglets born alive than typical of modern commercial operations. Furthermore, the milk quantity through weigh-suckle-weigh was not estimated. Other aspects of milk composition such as fat and carbohydrate were not measured. Each of these would have the potential to impact piglet growth but their impact in this study remains unknown. Additionally, whether the supplementations made relevant changes to the milk microbiome remains an open question. In a previous study, we reported that Oat supplementation of sows resulted in higher levels of bifidobacterium with probiotic potential [53]. Others report that yeast culture supplementation of sows led to increases in beneficial bacteria and suppression of opportunistic bacteria withing the gut of piglets [72]. Therefore, more investigation into the effect that these supplements make to sow milk quantity and quality is needed to gain better understanding of the impact they have on growth and incidences of PWD.

## 5. Conclusions

Incorporation of Oat into sow gestation and lactation feed, more than YC, resulted in heavier piglets at birth, decreased incidence of diarrhea and maintenance of weight post-weaning compared to YC. These results were associated with increases in immune and anti-bacterial milk components. These data together suggest that feeding sows a diet consisting of 15% oat during gestation and lactation may positively affect the intestinal health of offspring.

## Figures and Tables

**Table 1:** Ingredient composition of sow gestation diets.

Ingredient (%)	Control	YC	Oat	YC + Oat
Corn	81.30	80.90	66.33	66.00
Soybean Meal 48%	13.85	13.78	13.84	13.77
Limestone	1.11	1.11	1.11	1.11
MON-CAL 21% P	2.05	2.04	2.05	2.04
Salt	0.50	0.50	0.50	0.50
Vitamin Premix	0.15	0.15	0.15	0.15
Sow Vitamin Premix	0.04	0.04	0.04	0.04
Poultry Fat	1.00	1.00	1.00	0.99
Yeast Culture ^1^	-	0.50	-	0.50
Ground Whole Oat ^2^	-	-	14.99	14.91
Total Calculated composition ^3^	100	100	100	100
ME, kcal/kg	3300	3303	3174	3177
Crude Protein, %	13.3	13.4	13.7	13.9
Dry Matter, %	88.0	88.1	88.3	88.3
Starch, %	51.1	50.9	47.6	47.4
Amino acids				
Arginine, %	0.78	0.78	0.83	0.84
Histamine, %	0.37	0.37	0.37	0.37
Isoleucine, %	0.52	0.53	0.54	0.55
Leucine, %	1.28	1.28	1.26	1.26
Lysine, %	0.61	0.62	0.65	0.66
Methionine, %	0.24	0.24	0.31	0.32
Cystine, %	0.25	0.25	0.28	0.28
Tryptophan, %	0.14	0.14	0.15	0.15
Valine, %	0.62	0.62	0.66	0.66

^1^ Yeast culture (YC) was included at a rate of 5 g/kg; ^2^ whole ground oat included at a rate of 15% in place of corn. ^3^ Calculated using chemical composition data from Nutritional Research Council (NRC) (2012) Nutrient Requirements of Swine. ME = metabolizable energy.

**Table 2:** Ingredients composition of sow lactation diets.

Feed Ingredients (%)	Control	YC	Oat	YC + Oat
Corn	74.06	74.06	65.91	65.91
Soybean Meal 48%	17.62	17.62	10.86	10.86
Limestone Fine	1.08	1.08	1.08	1.08
MON-CAL 21% P	2.39	2.39	2.39	2.39
L-LYS 78%	0.16	0.16	0.16	0.16
Salt	0.50	0.50	0.50	0.50
Vitamin Premix	0.15	0.15	0.15	0.15
Sow Vitamin Premix	0.04	0.04	0.04	0.04
L-Threonine	0.01	0.01	0.01	0.01
Poultry Fat	4.00	4.00	4.00	4.00
Yeast Culture ^1^	-	0.50	-	0.50
Ground Whole Oat ^2^	-	-	14.97	14.97
Total	100	100	100	100
Calculated composition ^3^				
ME, kcal/kg	3436	3439	3317	3320
Crude Protein, %	14.7	14.8	12.4	12.6
Dry Matter, %	87.1	87.1	87.2	87.2
Starch, %	46.7	46.7	47.2	47.0
Amino acids				
Arginine, %	0.88	0.88	0.73	0.73
Histamine, %	0.40	0.40	0.33	0.33
Isoleucine, %	0.58	0.59	0.48	0.48
Leucine, %	1.35	1.35	1.14	1.15
Lysine, %	0.83	0.84	0.69	0.69
Methionine, %	0.25	0.25	0.29	0.29
Cystine, %	0.26	0.26	0.25	0.25
Tryptophan, %	0.16	0.16	0.13	0.13
Valine, %	0.67	0.68	0.59	0.59

^1^ Yeast culture (YC) was included at a rate of 5 g/kg; ^2^ whole ground oat included at a rate of 15% in place of corn. ^3^ Calculated using chemical composition data from Nutritional Research Council (NRC) (2012) Nutrient Requirements of Swine. ME = metabolizable energy.

**Table 3:** Composition of Piglet Grower Ration.

PIGLET GROWER RATION		
Feed Ingredient	Amount [kg]	% Inclusion
Corn meal	470.01	51.9
Wheat middlings	153.68	16.96
Hominy	136.08	15.0
HI-pro soybean meal	116.57	12.9
Ground limestone	10.89	1.2
Dist. dried grains	7.48	0.82
Phosphate mono/d	5.22	0.57
Salt (plain)	3.18	0.35
Lysine (78.8%)	0.91	0.1
Swine premix 2	0.91	0.1
Selenium premix (0.06%)	0.45	0.05
Feed preservative (65%)	0.45	0.05
**TOTAL**	905.82	100

**Table 4:** Immunoglobulin (Ig) Levels in Milk across Lactation.

Immunoglobulin Levels (mg/mL)				
**IgG**				
	**CON**	**YC**	**Oat**	**YC + Oat**
Day 0	13.2 ± 6.1 ^ab^	14.7 ± 2.1 ^a^	6.0 ± 3.1 ^b^	12.4 ± 5.2 ^ab^
Day 1	2.9 ± 1.0	7.9 ± 2.5	3.1 ± 0.9	5.0 ± 0.5
Day 4	1.9 ± 0.4	3.4 ± 1.4	2.9 ± 1.3	3.5 ± 0.5
Day 7	2.4 ± 1.1	3.5 ± 0.7	2.8 ± 2.0	5.0 ± 0.9
Day 14	3.2 ± 0.8	4.0 ± 0.4	3.7 ± 1.5	3.6 ± 1.1
**IgM**				
	**CON**	**YC**	**Oat**	**YC + Oat**
Day 0	6.7 ± 4.0	5.5 ± 3.1	4.6 ± 0.4	9.2 ± 5.4
Day 1	2.4 ± 0.9	2.8 ± 1.6	1.9 ± 0.6	2.9 ± 0.9
Day 4	1.6 ± 0.6	1.4 ± 0.3	1.9 ± 0.5	2.0 ± 1.2
Day 7	1.9 ± 0.5	2.2 ± 0.6	4.7 ± 1.1	2.9 ± 1.1
Day 14	1.9 ± 1.0	1.5 ± 0.3	3.3 ± 1.5	1.9 ± 0.5
**IgA**				
	**CON**	**YC**	**Oat**	**YC + Oat**
Day 0	3.2 ± 1.7	3.4 ± 1.9	4.6 ± 1.9	6.2 ± 2.8
Day 1	4.3 ± 1.3	6.8 ± 2.9	3.8 ± 1.3	5.2 ± 0.9
Day 4	1.7 ± 1.3	3.3 ± 1.8	1.7 ± 0.2	1.7 ± 0.7
Day 7	2.4 ± 1.1	3.5 ± 0.7	2.8 ± 2.0	5.0 ± 0.9
Day 14	3.2 ± 0.8	4.0 ± 0.7	3.7 ± 1.5	3.6 ± 1.1

Levels of immunoglobulin (Ig)A, IgG, IgA, and IgM within milk were measured by ELISA. Data are mean (*n* = 5). ± SD. ^a,b^ Differences between letters *a* and *b* are significant (*p* < 0.05).

**Table 5:** Gene ontology (GO) categories and KEGG pathways enriched by commonly expressed proteins (250) between control (CON), yeast culture (YC), and Oat sows on days 7 and 14 of lactation. FDR = false discovery rate.

GO Biological Process				
Term	Count	%	Benjamini	FDR
Platelet degranulation	26	12.7	3.95 × 10^−23^	4.16 × 10^−23^
Negative regulation of endopeptidase activity	18	8.8	4.18 × 10^−11^	8.81 × 10^−11^
GO Cellular Components				
Term	Count	%	Benjamini	FDR
Extracellular exosome	165	80.5	7.33 × 10^−98^	3.04 × 10^−97^
Extracellular space	106	51.7	5.99 × 10^−64^	4.98 × 10^−63^
Blood microparticle	42	20.5	2.67 × 10^−44^	3.33 × 10^−43^
KEGG Pathways				
Term	Count	%	Benjamini	FDR
Complement and coagulation cascades	11	5.4	4.08 × 10^−5^	2.70 × 10^−4^

**Table 6:** Quantitative proteomic analysis. Milk proteins differentially abundant between samples of sows fed CON, Oat or YC diets. Results are expressed as fold change of the difference in protein expression between the two treatments appearing in the heading. Cell color were assigned to label upregulated (red) and downregulated (blue) proteins between treatments.

Gene	Protein Names	CON-Oat	CON-YC	Oat-CON	YC-CON
BChE	Cholinesterase	−5.9	−5.4	5.9	5.4
SERPIND1	Serpin family D member 1	−5.5	−6	5.5	6
CHID1	Chitinase domain-containing protein 1 precursor	−5.4	−5.4	5.4	5.4
CES1	Carboxylic ester hydrolase	−0.9	−0.4	0.9	0.4
LTF	Lactoferrin	−0.8	−1.1	0.8	1.1
AHSG	Alpha-2-HS-glycoprotein	−0.8	−0.5	0.8	0.5
RAB7A	RAB7A; member RAS oncogene family	−0.4	−0.5	0.4	0.5
PPIC	Peptidyl-prolyl cis-trans isomerase	−0.1	0.3	0.1	−0.3
	Uncharacterized protein	0.3	−0.7	−0.3	0.7
ST13	Hsp70 Interacting Protein	1.5	1.7	−1.5	−1.7
CALM2	Calmodulin	1.6	1.6	−1.6	−1.6
RPLP0	60S acidic ribosomal protein P0	1.6	3.3	−1.6	−3.3
TUBA1A	Tubulin alpha chain	2	2.5	−2	−2.5
EIF5A	Eukaryotic translation initiation factor 5A	2.1	2.5	−2.1	−2.5
RPS7	40S ribosomal protein S7	2.6	3	−2.6	−3
VCP	Transitional endoplasmic reticulum ATPase	2.7	2.9	−2.7	−2.9
EEF2	Eukaryotic translation elongation factor 2	2.7	2.6	−2.7	−2.6
HSP90B1	Endoplasmin	2.7	3.7	−2.7	−3.7
PDIA3	Protein disulfide-isomerase	3.1	4.6	−3.1	−4.6
MDH2	Malate dehydrogenase; mitochondrial	3.3	4.1	−3.3	−4.1
CKB	Creatine kinase B-type	3.3	3.8	−3.3	−3.8
RPSA	40S ribosomal protein SA	3.3	3.7	−3.3	−3.7
RPLP2	60S acidic ribosomal protein P2	3.4	3.7	−3.4	−3.7
RACK1	Receptor of activated protein C kinase 1	3.4	4.6	−3.4	−4.2
ACTN4	Actinin alpha 4	3.7	2.1	−3.7	−2.1
SRM	Spermidine Synthase	3.7	3.8	−3.7	−3.8
HSP90AA1	Heat shock protein HSP 90-alpha	3.8	3	−3.8	−3
RNASE4	Ribonuclease 4	4.3	0.5	−4.3	−0.5
ATP5B	ATP synthase subunit beta	4.5	6.3	−4.5	−6.3
SERPINB1	Leukocyte elastase inhibitor	6.7	−0.2	−6.7	0.2

## Data Availability

All the raw LC-MS/MS data are submitted to MassIVE (massive.ucsd.edu/ (accessed on 22 January 2021)) with ID MSV000086562, and are publicly available.

## Supplementary Materials

The following are available online at https://www.mdpi.com/2076-2615/11/2/497/s1, Supplemental Table S1: Proteins commonly expressed between all the treatments; Supplemental Figure S1: Scatter plots showing the Pearson correlation coefficients of relative protein abundances (LFQ intensities) between biological replicates within the CON, OAT and YC experimental groups.
