# Supplementary material for: Inclusion of Oat and Yeast Culture in Sow Gestational and Lactational Diets Alters Immune and Antimicrobial Associated Proteins in Milk
Source: Animals (Basel). 2021 Feb 14;11(2):497. doi: 10.3390/ani11020497 (PMC7918739; doi:10.3390/ani11020497)
Supplement: Supplementary file 1 [file animals-11-00497-s001.pdf]

Table S1. List of proteins commonly expressed between the three treatments.

Protein ID	Gene names	Protein names	Average CON	Average Oat	Average YC
P04119	LGB	Beta-lactoglobulin-1A/1C (Beta-lactoglobulin IA/IC) (Beta-LG)	37.3068	37.6566	37.493
P39035	CSN1S1	Alpha-S1-casein	36.095	36.4877	36.3717
D5LG68	CSN2	Beta-casein (Fragment)	35.8591	36.2798	36.0822
P18137	LALBA	Alpha-lactalbumin (Lactose synthase B protein)	35.5458	35.8366	35.886
P11841	CSN3	Kappa-casein	35.4859	35.9168	35.672
A0A287AMK0	ALB	Serum albumin	35.1789	35.4702	35.7441
K7ZRK0	IGHA	IgA heavy chian constant region (Fragment)	34.6599	34.4079	35.0239
P00761	PRSS1	Trypsin (EC 3.4.21.4)	34.6533	34.8773	34.6735
P39036	CSN1S2	Alpha-S2-casein	34.5944	34.8817	35.1027
A0A286ZI90		Uncharacterized protein	34.5268	34.9893	34.967
B2CZF8	MFGE8	Milk fat globule-EGF factor 8 protein	33.3307	33.7306	33.9626
A0A0E3M2Q4	PIGR	Transmembrane secretory component poly-Ig receptor (Fragment)	33.1415	33.1526	33.3011
F1S3Y7	XDH	Xanthine dehydrogenase/oxidase	32.9137	33.0107	32.8503
A0A287A8U7		Uncharacterized protein	32.8188	32.5489	32.5839
F1RQB0	BTN1A1	Butyrophilin subfamily 1 member A1	32.7867	32.6616	32.6327
O02772	FABP3	Fatty acid-binding protein; heart (Fatty acid-binding protein 3) (Heart-type fatty acid-binding protein)	32.7333	32.4031	32.2953
L8B0W0	IGHG	IgG heavy chain	32.5679	31.857	32.4659
L8B180	IGHG	IgG heavy chain	32.5432	31.1521	31.8167
A0A287AE24		Uncharacterized protein	32.4237	31.8185	32.5389
O46655	WAP	Whey acidic protein (WAP)	32.0922	32.1834	32.6496
F1SQ51		Uncharacterized protein	31.9576	30.6963	30.5794
F1SBS4	C3	Complement C3	31.7664	31.3362	32.2335
Q6YT39	LTF	Lactotransferrin	31.744	32.2618	32.1739
L8B0R9	IGHG	IgG heavy chain	31.5331	29.3019	29.7677
A0A287BJL0	JCHAIN	Joining chain of multimeric IgA and IgM	31.3966	31.185	31.9273
A5YV76	FASN	Fatty acid synthase	31.0914	30.8073	29.707
K9J6H8	A2M	Alpha-2-macroglobulin	31.0697	30.8679	30.9446
A0A287AAR4	ACTB	Actin; cytoplasmic 1	30.7364	30.9314	31.2853
A0A287APD5	ACSL3	Long-chain-fatty-acid--CoA ligase 3	30.7059	30.8044	30.6451
A0A287AAY1	PLIN2	Perilipin	30.4749	30.6986	30.5295
F1SCC9	LOC106504545	Uncharacterized protein	30.4411	30.748	30.7745
H2EJJ3	Npt2B	Type IIb Na-dependent phosphate cotransporter	30.3977	30.4205	30.455
Q8SPS7	HP	Haptoglobin (Zonulin) [Cleaved into: Haptoglobin alpha chain; Haptoglobin beta chain]	30.3625	31.1163	31.9308
F1SS24	FN1	Fibronectin 1	30.3222	30.6437	30.2804
F1RRP2	LPO	Lactoperoxidase	30.3134	29.5586	29.2414
A0A287BGD6	ITIH4	Inter-alpha-trypsin inhibitor heavy chain H4	30.2942	29.507	30.0202
I3LJW2	FGG	Fibrinogen gamma chain precursor	30.2809	30.1403	30.2616
I3L651	FGB	Fibrinogen beta chain	30.2463	30.2134	30.2512
L8AXL9	IGHG	IgG heavy chain	30.1595	28.9678	29.4705
F1SCD0	LOC100153899	Uncharacterized protein	30.1097	29.6835	30.4886
Q6SVB3	MSTN	Myostatin	30.0116	30.1835	30.0163
F1RX36	FGA	Fibrinogen alpha chain	29.9227	29.9158	29.9445
P09571	TF	Serotransferrin (Transferrin) (Beta-1 metal-binding globulin) (Siderophilin)	29.911	29.6561	30.7686
A0A286ZI97	PGLYRP1	Peptidoglycan-recognition protein	29.8927	29.3545	30.2311
Q0QEM6	ATP5B	ATP synthase subunit beta (EC 3.6.3.14) (Fragment)	29.8792	25.375	23.5735
Q29549	CLU	Clusterin (CP40) (Complement cytolysis inhibitor) (CLI) [Cleaved into: Clusterin beta chain; Clusterin al	29.6974	28.165	28.9017
A0A287BHL7	ECM1	Extracellular matrix protein 1	29.6466	28.9416	29.5064
A0A287BM11	LOC396684	Uncharacterized protein	29.6447	29.1811	29.5933
I3LJC9	HAPLN3	Hyaluronan and proteoglycan link protein 3	29.5619	30.1667	30.0234
A5A8W8	C4A	Complement component 4A	29.4619	29.3151	29.7882
P49930	PMAP23	Antibacterial peptide PMAP-23 (Myeloid antibacterial peptide 23)	29.3828	28.0279	31.3424
L8AXK3	IGHG	IgG heavy chain	29.346	29.149	29.6749
P50390	TTR	Transthyretin (Prealbumin)	29.3186	29.2007	29.4517
F2Z5P1	H2AFV	Histone H2A	29.2814	29.1902	28.8484
L8B0U3	IGHG	IgG heavy chain	29.2619	29.9929	30.2298
F1RGR9	MUC1	Uncharacterized protein	29.2185	29.3339	29.4804
I3LEE6	PCOLCE	Procollagen C-endopeptidase enhancer	29.2009	29.0305	29.2379
A0A287AT48	LOC100153899	Uncharacterized protein	29.1971	28.6267	29.4333
A0A287BN06	PZP	Uncharacterized protein	29.0747	29.1232	29.0789
F1RYZ0	RPLP2	60S acidic ribosomal protein P2	29.0447	25.6933	25.3717
A0A286ZTC4		Uncharacterized protein	29.0413	28.5528	29.3179
Q6J9X9	LPL	Lipoprotein lipase (LPL) (EC 3.1.1.34)	29.0102	30.1559	30.1631
Q29594	CKB	Creatine kinase B-type (EC 2.7.3.2) (B-CK) (Creatine kinase B chain) (Creatine phosphokinase M-type) (	28.9858	25.6535	25.1385
Q29014	ORM1	Alpha-1 acid glycoprotein (Fragment)	28.9729	29.1702	28.993
A0A287A9T4	HSP90AB1	Heat shock protein HSP 90-beta	28.9657	27.4707	27.5231
A0A287ALS7		Uncharacterized protein	28.9335	28.7517	28.0661
A0A287B2M3	RPSA	40S ribosomal protein SA	28.9084	25.5686	25.2352
A0A287A1E0	EEF2	Eukaryotic translation elongation factor 2	28.8796	26.1417	26.2953
I3LN42	GC	GC; vitamin D binding protein	28.8586	29.0565	29.1559
K7GM40	APOA1	Apolipoprotein A-I	28.8545	30.2222	29.5673
A0A287AAD5	A1BG	Alpha-1B-glycoprotein	28.8334	29.578	29.1359
A0A287ALC1		Uncharacterized protein	28.7768	29.0712	29.2469
I3VKE6	CP	Ceruloplasmin	28.7433	28.7769	29.4176
F2Z5E2	SERPINC1	Antithrombin-III precursor	28.7089	28.9259	29.0187
A0A286ZUI3	EEF1A1	Elongation factor 1-alpha 1	28.6973	28.7863	28.4575
P50828	HPX	Hemopexin (Hyaluronidase) (EC 3.2.1.35)	28.6953	29.0957	29.3926
Q29092	HSP90B1 GRP94 T	Endoplasmin (94 kDa glucose-regulated protein) (GRP-94) (98 kDa protein kinase) (PPK 98) (ppk98) (H	28.6651	25.9228	24.9213
B9P414	SAA1	Serum amyloid A protein (Fragment)	28.6093	28.4192	28.7613
A0A287AJE3	LOC106504547	Uncharacterized protein	28.574	28.3502	28.7828
F1S9Q1	HSPA8	Heat shock cognate 71 kDa protein	28.5555	28.4239	28.1409
P20305	GSN	Gelsolin (Actin-depolymerizing factor) (ADF) (Brevin) (Fragment)	28.5517	28.3584	28.4141

D6PW25	MUC4	Mucin 4 transcript variant 4	28.5277	28.9271	28.5026
A0A287BG16	B4GALT1	Beta-1;4-galactosyltransferase 1	28.4894	28.4984	28.5946
F6QA08	PDIA3	Protein disulfide-isomerase (EC 5.3.4.1)	28.4823	25.3513	23.9273
P62936	PPIA	Peptidyl-prolyl cis-trans isomerase A (PPIase A) (EC 5.2.1.8) (Cyclophilin A) (Cyclosporin A-binding prot	28.4634	27.9314	27.8334
P29700	AHSG FETUA	Alpha-2-HS-glycoprotein (Fetuin-A) (Fragment)	28.46	29.274	28.921
I3LPB8	SRM	Uncharacterized protein	28.4583	24.7814	24.706
F1SCD1	SERPINA3-2	Alpha-1-antichymotrypsin 2 precursor	28.4536	28.5147	28.7153
A0A287BIL8	HSPA5	Endoplasmic reticulum chaperone BiP	28.4361	27.4144	26.8332
A0A287AVA9	LOC100153899	Uncharacterized protein	28.4256	28.5965	28.7764
Q0Z8R0	CST3	Cystatin	28.3059	28.6854	28.5044
Q7SIB7	PGK1	Phosphoglycerate kinase 1 (EC 2.7.2.3)	28.2657	27.4489	27.166
Q767L7	TUBB TUBB5	Tubulin beta chain (Tubulin beta-5 chain)	28.2347	26.1	25.4836
F1SIH8	VCP	Transitional endoplasmic reticulum ATPase	28.1948	25.4759	25.2693
P50447	SERPINA1 PI	Alpha-1-antitrypsin (Alpha-1 protease inhibitor) (Alpha-1-antiproteinase) (Serpin A1)	28.1707	28.3117	28.578
A0A287BGF7		Uncharacterized protein	28.1671	27.9856	28.0103
A0A287B7V5	TCN1	Transcobalamin-1	28.1516	27.541	29.0395
A0A287A7G8	HIST1H2BA	Histone H2B	28.1271	29.9794	28.4713
F1SHQ8	TUBA1A	Tubulin alpha chain	28.096	26.0565	25.5861
P00346	MDH2	Malate dehydrogenase; mitochondrial (EC 1.1.1.37)	28.0122	24.6993	23.9451
A0A287AIJ7	CD36	Platelet glycoprotein 4	28.0023	28.2754	28.0726
F1RII7	HBB	Hemoglobin subunit beta	27.9627	30.1138	27.1047
A0A287A4Q7	ACTN4	Actinin alpha 4	27.945	24.2786	25.8159
R4HZ39	XO	XDH xanthine dehydrogenase	27.9007	28.015	27.8758
K9IVH4	CPM	Carboxypeptidase M	27.8835	27.6673	28.3654
L8AXM5	IGHG	IgG heavy chain	27.85	27.0826	27.3343
Q9GMA6	SERPINA3-2	Alpha-1-antichymotrypsin 2	27.8459	28.1566	28.2579
P63246	RACK1 GNB2L1	Receptor of activated protein C kinase 1 (Guanine nucleotide-binding protein subunit beta-2-like 1) (F	27.844	24.4409	23.6881
I3LK59	ENO1	Enolase 1	27.7662	27.601	27.7881
P62802	HIST1H4A	Histone H4	27.7585	29.4141	28.4556
G9F6X8	P4HB	Protein disulfide-isomerase (EC 5.3.4.1)	27.7437	25.7711	25.512
O02705	HSP90AA1 HSP90/	Heat shock protein HSP 90-alpha	27.7348	23.9393	24.7341
P68137	ACTA1 ACTA	Actin; alpha skeletal muscle (Alpha-actin-1) [Cleaved into: Actin; alpha skeletal muscle; intermediate f	27.7122	27.5453	28.0854
K7GNX7	SLC6A14	Transporter	27.5922	27.4429	27.9209
Q29545	ICA	Inhibitor of carbonic anhydrase	27.5899	27.984	27.9271
F1RFY1	PFN1	Profilin	27.5751	27.3775	27.7527
A0A287B356	EIF5A	Eukaryotic translation initiation factor 5A (eIF-5A)	27.5484	25.4103	25.0407
F1S682	QSOX1	Sulfhydryl oxidase (EC 1.8.3.2)	27.5469	27.7083	27.8137
Q29214	RPLP0	60S acidic ribosomal protein P0 (60S ribosomal protein L10E)	27.5381	25.9334	24.2209
P08059	GPI	Glucose-6-phosphate isomerase (GPI) (EC 5.3.1.9) (Autocrine motility factor) (AMF) (Neuroleukin) (NL	27.5295	26.2835	26.3979
I3L5B2	RPS7	40S ribosomal protein S7	27.4906	24.8829	24.4868
P79403	GANAB	Neutral alpha-glucosidase AB (EC 3.2.1.84) (Alpha-glucosidase 2) (Glucosidase II subunit alpha)	27.4567	26.7571	26.1521
A0A286ZW70	PPIB	Peptidyl-prolyl cis-trans isomerase (PPIase) (EC 5.2.1.8)	27.455	27.4649	26.9129
A0A287B5Y6	LBP	Lipopolysaccharide-binding protein precursor	27.3583	27.6285	27.8411
F1RK02	LCP1	Lymphocyte cytosolic protein 1	27.3443	24.9529	26.4132
A0A287A2R9	YWHAE	Tyrosine 3-monooxygenase/tryptophan 5-monooxygenase activation protein epsilon	27.3025	26.8608	26.6931
Q9GLW8	PRDX5	Peroxiredoxin 5	27.2878	26.5691	26.0942
F1SD69	LGMN	Legumain	27.2578	27.1474	26.5981
F1SVA2	TINAGL1	Tubulointerstitial nephritis antigen like 1	27.2566	27.6204	27.8142
L8B0S2	IGHG	IgG heavy chain	27.2149	27.9221	27.5906
Q2EN76	NME2	Nucleoside diphosphate kinase B (NDK B) (NDP kinase B) (EC 2.7.4.6) (Histidine protein kinase NDKB) (	27.2119	26.9854	26.6611
P01965	HBA	Hemoglobin subunit alpha (Alpha-globin) (Hemoglobin alpha chain)	27.1888	29.415	26.4201
Q95274	TMSB4	Thymosin beta-4 (T beta-4) [Cleaved into: Hematopoietic system regulatory peptide (Seraspenide)]	27.1595	25.4929	27.3678
Q0Z8U2	RPS3	40S ribosomal protein S3 (EC 4.2.99.18)	27.1448	26.1593	25.6944
Q9GMA7	SERPINA3-1	Alpha-1-antichymotrypsin 1 (Fragment)	27.1341	27.1076	27.4127
P80310	S100A12	Protein S100-A12 (Calgranulin-C) (CAGC) (Extracellular newly identified RAGE-binding protein) (EN-RA	27.1196	26.5488	28.4091
A0A287AA42		Uncharacterized protein	27.1103	27.2955	27.0008
F2Z5G3	CALM2 CALM1 CA	Calmodulin	27.1034	25.5106	25.5448
F1SFZ5	MUC15	Mucin 15; cell surface associated	27.079	27.3099	27.5637
P00355	GAPDH GAPD	Glyceraldehyde-3-phosphate dehydrogenase (GAPDH) (EC 1.2.1.12) (Peptidyl-cysteine S-nitrosylase G	27.0694	28.0329	27.7516
A0A287A8T0	RPL7	60S ribosomal protein L7	27.0151	24.9658	24.0535
Q4R0H6	PSP-I	Spermadhesin PSP-I	27.0014	27.6705	27.4282
A0A287A8P0	OS9	OS9; endoplasmic reticulum lectin	26.9877	26.9007	27.102
A0A286ZLR0		Uncharacterized protein	26.9793	26.6929	27.7208
A0A287B0L6	LOC110261668	Histone H2A	26.9792	28.809	27.3086
P18650	APOE	Apolipoprotein E (Apo-E)	26.9777	27.4923	27.1532
D0G7F6	TPI1	Triosephosphate isomerase (EC 5.3.1.1)	26.964	25.5668	25.4856
Q95ME5	SOD1	Superoxide dismutase 1 (Fragment)	26.9404	26.1285	25.2964
K4P7U5	PLIN3	Perilipin	26.9157	26.5311	26.3511
F1RUK8	GDI2	Rab GDP dissociation inhibitor	26.8792	26.9659	26.7545
A0A287AE76	RPL4	60S ribosomal protein L4	26.8534	25.3161	24.6087
C3S7K6	S100A9	Calcium-binding protein A9 (Calcium-binding protein S100A9) (RNA-binding region containing protein	26.8268	25.6639	28.3612
F1SJT7	APOA4	Apolipoprotein A-IV	26.8241	27.761	27.1764
A0A287B8B1	ST13	Uncharacterized protein	26.824	25.2938	25.0957
F1SS26	THBS1	Thrombospondin-1 precursor	26.8097	26.9015	26.883
I3LED4	CIDEA	Cell death activator CIDE-A	26.8077	26.7382	26.3781
P14287	SPP1 OPN	Osteopontin (Bone sialoprotein 1) (Secreted phosphoprotein 1) (SPP-1)	26.7879	26.9274	26.9021
F1RIP6	NUCB1	Nucleobindin-1 precursor	26.7711	26.6649	26.7904
F1SB42	EZR	Ezrin	26.7555	26.7537	26.5056
F1RKG8	PEBP1	Uncharacterized protein	26.7511	24.5022	25.0256
A1XQU9	RPS20	40S ribosomal protein S20	26.6919	25.2149	24.6845
Q8WMN8	LTF	Lactoferrin (Fragment)	26.6823	27.498	27.7766
A0A286ZYX8	ALDOA	Fructose-bisphosphate aldolase (EC 4.1.2.13)	26.6601	27.2662	27.0442
U5NEE3	ANPEP	Aminopeptidase (EC 3.4.11.-)	26.6524	26.3822	26.4351

A8U4R4	tkt TKT	Transketolase	26.636	25.0438	25.4025
B8XH67	SLC9A3R1	Na(+)/H(+) exchange regulatory cofactor NHE-RF	26.6337	26.6795	26.3306
F1SC82	MYOF	Myoferlin	26.6075	26.6321	26.5676
F2Z558	YWHAZ	14-3-3 protein zeta/delta	26.5252	26.1146	26.016
F1RJF7	SDF4	Stromal cell derived factor 4	26.5007	25.9038	26.4725
P10668	CFL1	Cofilin-1 (Cofilin; non-muscle isoform)	26.4621	26.2177	26.459
F1S9A4	NUCB2	Uncharacterized protein	26.4468	25.844	25.8771
B9TSR8	CFB	Complement factor B	26.3892	26.9451	27.1809
A0A286ZVC3	AZGP1	Uncharacterized protein	26.3866	26.3604	25.9726
F1RGC4	LOC100519082	Uncharacterized protein	26.3495	26.3717	27.0687
I3L728		Uncharacterized protein	26.2703	25.6086	26.5908
P19620	ANXA2 ANX2	Annexin A2 (Annexin II) (Annexin-2) (Calpactin I heavy chain) (Calpactin-1 heavy chain) (Chromobindir	26.2438	26.442	26.6838
E7EI20	ARHGDIA	Rho GDP dissociation inhibitor alpha (Rho GDP-dissociation inhibitor 1)	26.1999	25.396	25.5366
K7GQ50	S100A1	Protein S100 (S100 calcium-binding protein)	26.1538	25.8096	25.1473
F1S3U9	PRDX1	Uncharacterized protein	26.0795	26.9809	25.709
F2Z5B1	SERPINB1	Leukocyte elastase inhibitor (Serpin peptidase inhibitor; clade B (Ovalbumin); member 1 tv1)	26.0661	19.4114	26.27
F1RUM4	ITIH2	Inter-alpha-trypsin inhibitor heavy chain H2	26.0455	26.2939	26.6203
Q28944	CTSL CTSL1	Cathepsin L1 (EC 3.4.22.15) (Cathepsin L) [Cleaved into: Cathepsin L1 heavy chain; Cathepsin L1 light c	26.0308	25.6073	25.7154
F1SHL9	PKM	Pyruvate kinase (EC 2.7.1.40)	25.9739	28.061	27.3861
F1SUP9	CHRDL2	Chordin like 2	25.9405	26.1571	25.8337
A0A287AJI4	SERPINF2	Serpin family F member 2	25.9034	26.676	26.4906
A0A286ZFW3	APOH	Apolipoprotein H	25.8733	26.0574	26.4842
F1SRC8	CLEC3B	C-type lectin domain family 3 member B	25.872	26.0618	26.1672
F6Q5P0	RPS13	40S ribosomal protein S13	25.8664	25.027	24.5681
A0A286ZIL9	COL18A1	Collagen type XVIII alpha 1 chain	25.85	25.6967	26.0986
Q76L24	TLR-2	Toll-like receptor 2	25.7835	24.9132	25.6061
A0A287AS29	NME1	Nucleoside diphosphate kinase (EC 2.7.4.6)	25.7338	25.5665	24.4222
C4MXZ1	ARF1	ADP-ribosylation factor 1	25.7202	26.1204	25.717
A0A287ATE3		Uncharacterized protein	25.6854	25.7189	25.6778
P02543	VIM	Vimentin	25.6172	26.306	26.1863
P48819	VTN	Vitronectin (VN) (S-protein) (Serum-spreading factor)	25.5002	25.9245	25.932
F1RFQ7	RAN	GTP-binding nuclear protein Ran	25.4942	24.9326	24.3001
A0A287AHC1		Uncharacterized protein	25.4747	24.7337	25.4321
F1SGQ0	DHRS1	Dehydrogenase/reductase 1	25.4418	25.2472	24.9388
I3LC07	RAB18	RAB18; member RAS oncogene family	25.4367	25.7799	25.6238
Q9GKJ6	BCHE	Carboxylic ester hydrolase (EC 3.1.1.-) (Fragment)	25.4176	26.358	25.8295
F1S7K2	LRG1	Leucine rich alpha-2-glycoprotein 1	25.3562	25.7023	25.8724
F1SJW8	SERPING1	Plasma protease C1 inhibitor precursor	25.3232	26.3207	25.9315
F1SJB5	ANXA1	Annexin	25.3228	25.0788	27.2666
F1SMC0	CAP1	Adenylyl cyclase-associated protein	25.2912	25.0744	24.6769
F1S1G8	LOC110256000	Amine oxidase (EC 1.4.3.-)	25.2873	25.516	25.9271
F1STN0	SMPDL3B	Sphingomyelin phosphodiesterase acid like 3B	25.2699	26.2158	26.321
A0A287ALQ2	ACSL6	Uncharacterized protein	25.2397	24.9314	24.0303
F2Z4Z1	YWHAG	Uncharacterized protein	25.2292	25.0157	24.4907
K9J6I5	ERAP1	Aminopeptidase (EC 3.4.11.-)	25.1916	25.5946	25.3117
A0A287AJQ2	PGAM1	Phosphoglycerate mutase (EC 5.4.2.11) (EC 5.4.2.4)	25.1905	25.5013	24.8936
P27917	APOC3	Apolipoprotein C-III (Apo-CIII) (ApoC-III) (Apolipoprotein C3)	25.1719	25.1441	25.3335
Q6S4N2	HSPA1B	Heat shock 70 kDa protein 1B (Heat shock 70 kDa protein 2) (HSP70.2)	25.1715	24.5159	23.8379
L8B130	IGHG	IgG heavy chain	25.1183	25.2615	25.0578
K9J4V7	CSF2RA	Granulocyte-macrophage colony-stimulating factor receptor subunit alpha	25.1116	25.125	25.3142
F1RPA3	GPRC5B	G protein-coupled receptor class C group 5 member B	25.1008	24.9343	24.7509
P06867	PLG	Plasminogen (EC 3.4.21.7) [Cleaved into: Plasmin heavy chain A; Activation peptide; Plasmin heavy ch	25.0168	25.5615	25.5839
F1SH96	ITIH1	Inter-alpha-trypsin inhibitor heavy chain H1	25.0147	25.7877	25.7307
A0A287B2P1	CLIC1	Chloride intracellular channel protein	24.9953	24.5839	24.6461
Q2VTP6	FKBP1A	Peptidyl-prolyl cis-trans isomerase FKBP1A	24.9254	24.2267	24.4423
K7GLN4	PRDX4	Peroxiredoxin 4	24.9234	25.6508	25.0767
P81245	OBP	Odorant-binding protein (OBP)	24.8683	24.8435	25.045
Q29243	DAG1	Dystroglycan (Dystrophin-associated glycoprotein 1) [Cleaved into: Alpha-dystroglycan (Alpha-DG); Be	24.8016	24.5255	24.9553
K7GPW1	CFI	Uncharacterized protein	24.7841	25.0099	25.4479
I3LSK5	GNB1	G protein subunit beta 1	24.756	25.0651	24.895
P51779	CFD DF	Complement factor D (EC 3.4.21.46) (Adipsin) (C3 convertase activator) (Properdin factor D)	24.7325	24.5027	24.8578
A0A286ZP69	RAB7A	RAB7A; member RAS oncogene family	24.6556	25.0158	25.1243
F1RGI9	SIL1	SIL1 nucleotide exchange factor	24.5292	25.562	25.1777
A0A287AP83	EGF	Pro-epidermal growth factor	24.5164	24.2775	24.8536
A0A287B931	RAP1B	RAP1B; member of RAS oncogene family	24.5066	24.7856	24.5679
A0A287B2U6	SNAP23	Synaptosomal-associated protein	24.4523	24.2801	24.2094
A0A287BEC3	CEACAM1	Uncharacterized protein	24.4224	24.3652	24.3061
Q0PM28	SERPINF1	Pigment epithelium-derived factor precursor	24.4093	25.0997	25.0205
Q1KS52	ALS IGFALS	Acid-labile subunit (Insulin-like growth factor-binding protein complex acid labile subunit precursor)	24.3864	25.2772	24.9414
F1RG45	AGT	Angiotensinogen	24.3304	25.2623	25.5033
A0A287AH59	STX3	Syntaxin 3	24.2837	24.3082	24.4667
A0A287B1C7	LMNA	Prelamin-A/C	24.2098	24.6806	24.1788
A0A287AEA2	PPIC	Peptidyl-prolyl cis-trans isomerase (PPIase) (EC 5.2.1.8)	24.2062	24.2906	23.9035
K9IWA3	ATP6AP1	ATPase H+ transporting accessory protein 1 (V-type proton ATPase subunit S1)	24.2055	24.5896	24.7166
D5FGE4	GLA	Alpha-galactosidase (EC 3.2.1.-)	24.1773	25.0851	24.7775
A0A287AZG3	MAN2A1	Alpha-mannosidase (EC 3.2.1.-)	24.1679	24.1923	24.0669
F1RK01	CPB2	Carboxypeptidase B2	24.1006	24.2001	24.3039
F1SFI6	FETUB	Fetuin B	24.0992	24.9613	23.9473
A0A286ZNI7	PRSS8	Serine protease 8	24.0725	23.8023	23.7223
K7GQE8	LOC110255210	Uncharacterized protein	24.0578	24.301	24.0272
A0A287BDU7	PZP	Uncharacterized protein	24.0137	24.1926	23.9522
P04366	AMBP ITIL	Protein AMBP [Cleaved into: Alpha-1-microglobulin; Inter-alpha-trypsin inhibitor light chain (ITI-LC) (E	23.9544	24.2207	23.875
F1RS82	BTD	Biotinidase	23.8654	23.8784	23.9625

Q15KJ1	CD14	Monocyte differentiation antigen CD14 (Myeloid cell-specific leucine-rich glycoprotein)	23.7918	23.67	24.8238
F1RT83	SDCBP	Syntenin-1 CG9291-PB	23.7699	23.9644	24.0571
P15468	RNASE4 RNS4	Ribonuclease 4 (RNase 4) (EC 3.1.27.-) (Ribonuclease PL3)	23.7529	19.4114	23.2558
F1RUM1	AFM	Afamin	23.5209	24.0819	23.8525
F1SI88	HEXA	Beta-hexosaminidase (EC 3.2.1.52)	23.2996	23.511	23.4572
F1RKY2	SERPIND1	Serpin family D member 1	19.4114	24.9568	25.3683
F1RZ01	CHID1	Chitinase domain-containing protein 1 precursor	19.4114	24.8117	24.783
A0A287AX20	BCHE	Cholinesterase	19.4114	25.3548	24.7783

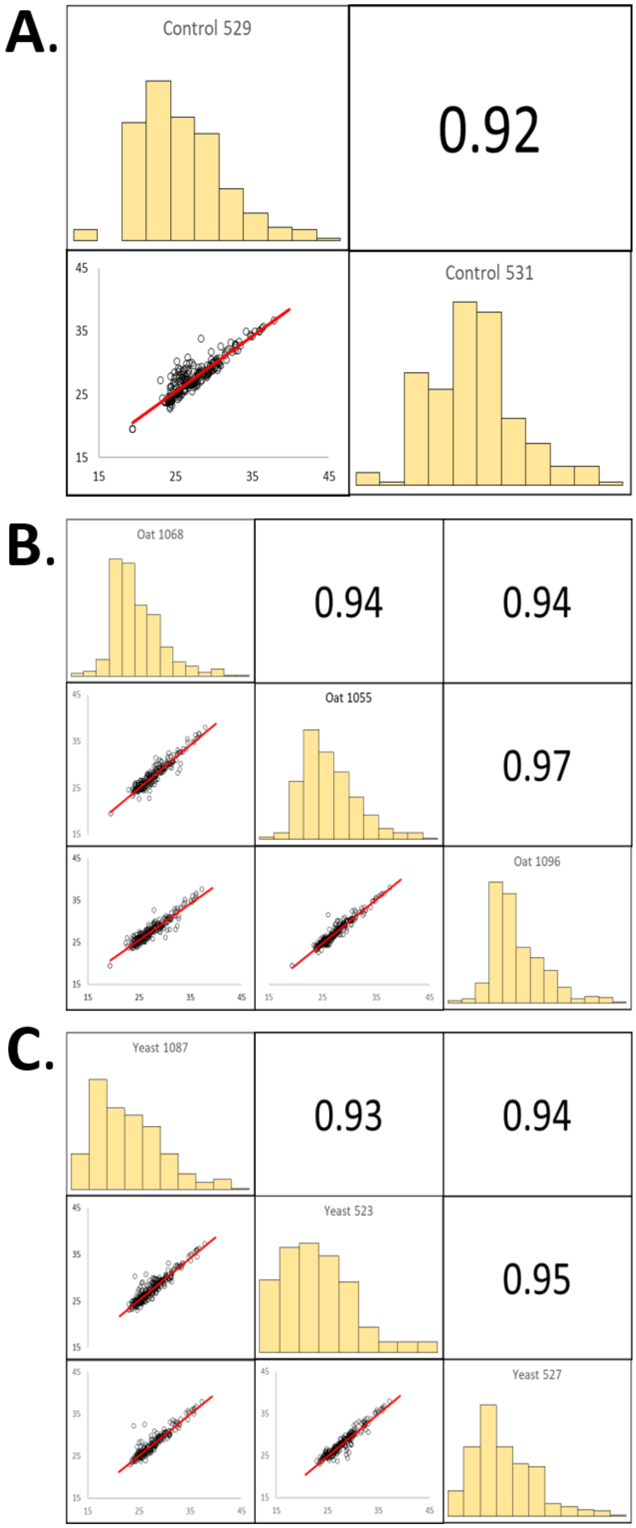

Supplemental Figure S1. Scatter plot showing the Pearson correlation coefficients between biological replicates and histograms for individual samples, of relative protein abundances (LFQ intensities) within the (A) Control, (B) Oat, and (C) Yeast experimentals group.
